# Biopsychosocial aetiologies of gender-based violence among convicted South African men: a narrative literature review

**DOI:** 10.1108/IJOPH-04-2025-0034

**Published:** 2026-11-17

**Authors:** Themba Fotrick Mavasa, Wandile Tsabedze, Lindokuhle Mdeyi Ubisi

**Affiliations:** Department of Psychology, University of South Africa, Pretoria, South Africa

**Keywords:** Biopsychosocial framework, Aetiologies, Gender-based violence, Convicted men, Management of risk, Risk assessment

## Abstract

**Purpose:**

The phenomenon of gender-based violence (GBV) is a global concern. Neither women nor men are immune to GBV. However, many mental health workers in forensic and non-forensic settings work closely with men convicted of GBV offences in need of assessment and the management of violent behaviour. As such, it is imperative to uncover the biopsychosocial aetiologies of GBV among convicted South African men who perpetrated GBV.

**Design/methodology/approach:**

A desktop narrative review of available qualitative and quantitative studies from 2015 to2025 was conducted to search for evidence. Literature was analysed thematically, using a hybrid deductive inductive approach in which the three domains of the biopsychosocial model served as predetermined themes. The review was grounded in Roy Grinker’s (1952) concept of a biopsychosocial model, which was expanded upon and underpinning the framework in George Engel’s (1977) model.

**Findings:**

Sub-themes identified in the literature were grouped under three predetermined themes derived from the biopsychosocial framework: sociocultural, psychological and biological factors. South Africa’s legacy of colonialism and apartheid emerged as the overarching context within which psychological and biological risk factors operate.

**Research limitations/implications:**

This study findings primarily pertain to men in South Africa and may not be generalisable to other settings. While data show men are often perpetrators of GBV, these figures must be viewed in a systemic context, considering issues like crime data that are subject to limitations, including underreporting. While the literature provides valuable insights into the biological factors associated with GBV from international studies, there remains a notable gap in critically synthesising the biological factors’ findings to the South African context.

**Originality/value:**

The authors concluded and recommended the provision of psychologists and other healthcare service providers in forensic and non-forensic settings, such as probation officers, police officers and social services officials, in conducting risk assessment, management of risk of men convicted of GBV offences, as well as early reduction interventions.

## Introduction

Gender-based violence (GBV) is a global concern (Tappis *et al.*, 2016). It is described as acts of violence committed against an individual or group of individuals due to their gender. It includes behaviours that have the potential to cause physical, psychological, sexual, economic or emotional harm to another person, resulting from gendered power inequalities (Yaliwe *et al.*, 2013). In general, GBV refers to violent acts, threats, force or denial of basic human rights, whether committed publicly or privately, that cause physical, psychological or sexual harm to a person (United Nations, 1993; Modise and Modise, 2023; Deane, 2024). Both men and women can experience violence or be perpetrators of violence. However, women are more likely to experience violence than men (Arango *et al.*, 2014; Oosthuizen *et al.*, 2024). By a very significant margin, most violence, including violent crime, is committed by males (Golding and Fitzgerald, 2019). Starting in preschool and elementary school, boys are more likely to be disciplined and suspended for conduct problems, as they commonly display problem behaviours and exhibit antisocial features more frequently than girls (Golding and Fitzgerald, 2019; Jacob, 2022). By adolescence, the juvenile arrest rate for boys for violent crimes is four times higher than that for girls (Golding and Fitzgerald, 2019). In adulthood, the rate of males committing violent crimes is also about four times more than that of women (Golding and Fitzgerald, 2019). The numbers of those arrested for the most violent crimes (murder, rape and robbery) are even higher for males (88%) (Golding and Fitzgerald, 2019).

Violence has been part of the South African social context for decades, rooted in historical apartheid policies and underpinned by high levels of inequality and poverty, racism, unequal gender power relations, hostility to sexual and gender diversity and the legacies of colonialism and ongoing structural oppressions (Government of South Africa, 2020; Van Niekerk and Boonzaier, 2015). The country’s history of colonialism and apartheid, and racialised policies implemented is said to have shaped gender relations and operated as a “master narrative” in mapping out the course of men’s subjectivities and the ways they defined their gender identities, how these played out in daily life and how they impacted the construction of contemporary masculinities (Pyke, 2020). In modern South Africa, the male identity is still strongly tied to ownership of, or stewardship over, a woman and children. Without a wife and children, an adult man is still considered a boy. This cultural prerequisite to manhood conditions men to view women as something to be obtained and controlled rather than autonomous individuals, which emphasises the idea that women are weaker and should be subordinate (George, 2020).

## Brief literature

### Prevalence of GBV in the Southern African region

In recent years, South Africa has faced extreme levels of violence and GBV (Graaff and Heinecken, 2017). South Africa continues to have some of the most comprehensive and progressive laws, policies and programmes aimed at addressing GBV (Enaifoghe and Idowu, 2021). However, the existence of progressive legislation has not translated into a reduction in GBV; despite the numerous laws, policies and programmes developed since 1994, the problem appears to be worsening (Department of Higher Education and Training, 2020). The protection these laws offer therefore depends on how they are applied within the criminal justice system, which involves several key departments and institutions that work together to uphold the law and ensure justice (South African Government, 2026). The criminal justice system is a convenient arrangement of a number of state-run bureaucratic institutions such as South African Police Service (SAPS), National Prosecuting Authority (NPA), the Judiciary (Judges and Magistrates), Department of Justice and Constitutional Development, and Department of Correctional Services (DCS) that deal with offending and offenders (Daly and Sarre, 2017; South African Government, 2026). The SAPS Crime Statistics report for the second quarter of 2022 / 23 showed that between July and the end of September 2022, the murder of women worsened in the second quarter, with 989 women murdered. Between July and September 2022, 10,590 rape cases were reported nationally, compared to 9,556 the previous year. More than 13,000 women were also victims of assault with the intent to cause grievous bodily harm, and 1,277 women were victims of attempted murder (Department of Correctional Services, 2023). In the reporting of GBV statistics for the period between July and September 2022, the SAPS Family Violence, Child Protection and Sexual Offences units arrested more than 4,000 alleged perpetrators of GBV, and 410 alleged rapists were traced and arrested. More than 17,000 trial-ready GBV cases were processed by teams of the SAPS and the National Prosecuting Authority (State of the Nation, 2022).

Although many mental health workers in forensic and non-forensic settings, as well as professionals from other disciplines such as probation officers, police officers and social services officials, work closely with those convicted of GBV offences in need of assessment and management of violent behaviour; however, GBV offenders tend to have high rates of recidivism (Yakeley, 2017; Winner, 2023). By December 2022, South Africa had a total sentenced inmate population of approximately 514,723 and an average of 51,919 remand detainees in its 243 correctional centres (Department of Correctional Services, 2023). According to George (2020), in a study conducted by the South Africa Medical Research Council (MRC) in 2009, 27.6% of men interviewed admitted to raping a woman or girl. Additionally, 46.3% of those men admitted repeating offences, and 7.7% of self-admitted repeat offenders reported raping 10 or more women.

The literature points to sociocultural, psychological and biological sources of GBV in South Africa. At the sociocultural level, the apartheid system, patriarchal culture, gender norms, poverty and neighbourhood disorganisation and crime all contribute to an ecology of risk for higher levels of GBV (Government of South Africa, 2020; Graaff and Heinecken, 2017; Corvo, 2019). In South Africa, GBV is driven largely by gender inequalities and by norms that define masculinity as dominance over women, aggressiveness and sexual conquest (Van den Berg *et al.*, 2013; Peacock, 2013). At the psychological level, predispositions to GBV are rooted in individual psychopathology or dysfunctional personality structures shaped by early childhood experiences (Grover, 2015), including early-life exposure to maltreatment and intrafamilial violence, insecure attachment and difficulties with mentalising and emotional regulation (Corvo, 2019; Taubner *et al.*, 2017; Berke *et al.*, 2019); violence is thus also a learnt behaviour (Treves-Kagan *et al.*, 2020). At the biological level, genetic vulnerabilities, neurotransmitters such as serotonin and dopamine and brain injury have been implicated (Ali and Naylor, 2013; Chester and DeWall, 2018; Corvo and Dutton, 2015; Rogers and Ali, 2023). These factors do not operate in isolation, men’s perpetration of violence against women results from a complex, interconnected ecology of sociological, psychological and biological factors which reciprocally influence each other (Fleming *et al.*, 2015; Ali and Naylor, 2013; Lehman *et al.*, 2017).

Considering that violence against women has complex and multifaceted root causes, prevention strategies must be ambitious and holistic (Peacock, 2013). Furthermore, it is clear that GBV is a multifaceted phenomenon reciprocated by biological, psychological and sociocultural dynamics that unfold over personal and historical time, which need to be considered by policymakers, researchers and health professionals when intervening and/or developing interventions for men convicted of GBV offences (Meer, 2013; Lehman *et al.*, 2017; Peacock and Barker, 2014).

## Theoretical orientation

The biopsychosocial model served as a central component of the theoretical framework for this study (Lugg, 2022). The review was grounded in Roy Grinker’s (1952) concept of a biopsychosocial model, which was expanded upon and underpinning the framework in George Engel’s (1977) model. According to Lugg (2022), the biopsychosocial (BPS) model remains the predominant theoretical framework underpinning contemporary psychiatric training and practice. Each factor or component of the biopsychosocial model includes systems that reciprocally influence other dynamics in the model (see Figure 1), and some factors are particularly intertwined; therefore, it affects behaviour (Lehman *et al.*, 2017). The following are illustrations of the biopsychosocial model.

**Figure 1:**
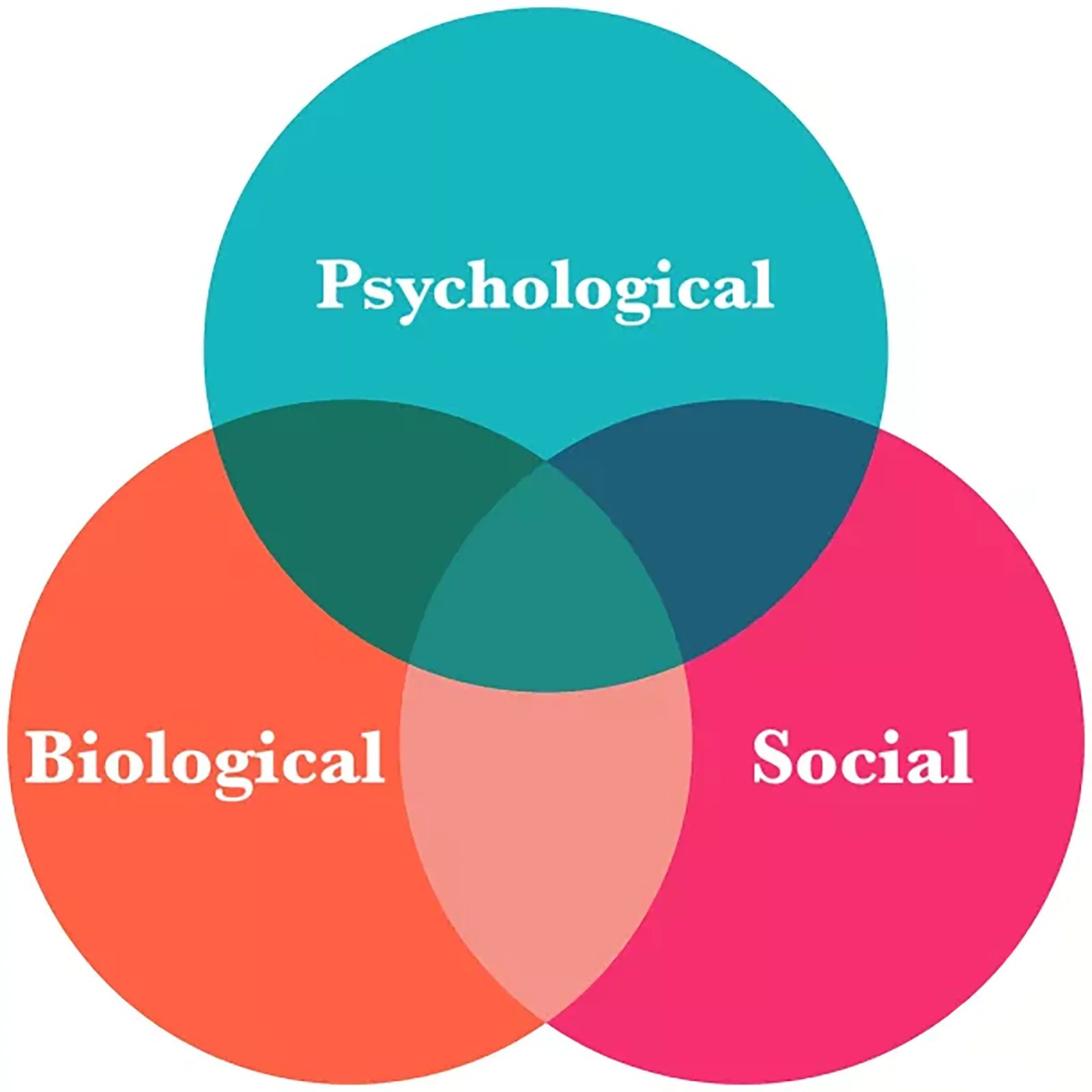
Diagram of the biopsychosocial model

According to Lehman et al. (2017), biological factors capture the physical elements of the body that affect and determine behaviour. Biological dynamics are important for understanding how the psychological and physical domains affect behaviour (Lehman *et al.*, 2017). Each of these systems, by itself, is formed by a complex, interconnected set of structures and cells. For example, gene–environment interactions, social genomics and epigenetics contributed to propelling scientific understanding of the reciprocal role of biological factors in understanding social and psychological dynamics (Lehman *et al.*, 2017). Lehman et al. (2017) also indicate that psychological factors include cognitive, emotional, motivational, attitudinal and behavioural systems that affect behaviour. Psychological factors focus on the role of the self, identity, personality, coping strategies, substance use and addiction, stress appraisal, emotions, illness schemas, anxiety and depression and health behaviour in understanding behaviour (Lehman *et al.*, 2017), while social factors are divided into interpersonal factors and broader contextual dynamics (e.g. culture) (Lehman *et al.*, 2017).

Wright et al. (2019) maintain that the biopsychosocial model’s conceptual work explains how biological, sociological and psychological factors interact and contribute to the aetiology and maintenance of individual behaviour through the application and combination of a well-known model (i.e. the 3P model). The 3P model (predisposing, precipitating and perpetuating factors) include factors that provide a more robust, and importantly, a time-inclusive argument of how different factors may manifest and subsequently accumulate to propel an individual up the “ladder” and over the “threshold” of an individual’s state of behavioural manifestation (Wright *et al.*, 2019).

Unlike earlier theoretical formulations of intergenerational transmission of domestic violence as only learnt behaviour produced by exposure to parental violence (Corvo, 2019), a biopsychosocial framework acknowledges its aetiological complexity by emphasising the role of psychological and social factors, in addition to biological factors in human behaviour (Wright *et al.*, 2019). Biological, psychological and social influences are important in understanding offending behaviour and coordinating methods to support change (Walton *et al.*, 2017). In this review, the three domains of the model were therefore used as predetermined (*a priori*) themes, under which the sub-themes identified in the literature were organised. This review aimed to answer the question: “What are the reported biopsychosocial aetiologies of GBV among convicted South African men?”

## Methods

A desktop narrative review was used to peruse the literature in this area. The narrative review approach was employed to synthesise existing literature on the biopsychosocial aetiologies of GBV among convicted men in South Africa. The aim was to generate a comprehensive understanding of the multifaceted factors contributing to GBV within this specific context, guided by the biopsychosocial framework as an organising model. Given the complexity and interdisciplinary nature of the research question, a narrative review was deemed appropriate to integrate diverse types of evidence ranging from qualitative, quantitative and mixed-methods studies to develop a nuanced, contextualised understanding of the phenomenon. Unlike systematic reviews, which focus on evaluating and synthesising evidence following strict inclusion criteria, a narrative review allows for flexibility in source selection and an interpretive synthesis, which is suited for exploring complex, multifaceted issues such as GBV within a specific sociocultural context. While not a formal systematic review, this exploratory search was used to identify representative high-quality sources of evidence from any existing literature from all study designs related to the phenomenon being studied. The findings were woven into a cohesive narrative, providing an overview of the study design in relation to biopsychosocial aetiologies of GBV among convicted South African men. Emphasis was placed on highlighting important advancements, areas of consensus, ongoing debates within the field and gaps in the literature. By adopting this approach, the reviewer aimed to provide a broad and insightful synthesis of the literature on biopsychosocial aetiologies of GBV among convicted South African men, contributing to the understanding of its complexities and guiding future research and clinical practice.

## Search strategy

To compile this desktop narrative review, the reviewer conducted a preliminary, illustrative search across several major databases, including PsycInfo, EBSCOhost, Google Scholar, JSTOR, PubMed, Scopus, Sabinet, PsychNet, Provincial and Governmental websites, covering the period between 2015 and 2025.

## Selection criteria

The search for both qualitative and quantitative publications used certain keywords, such as “gender-based violence”, “violent behaviour”, “aetiologies of gender-based violence”, “biopsychosocial factors”, “men and violent behaviour”, “convicted South African men”, “biopsychosocial aetiologies of GBV”, “GBV and men” and other variants of similar key terms. Reference lists from consulted publications were followed to snowball for more publications. Due to the scope and word limit of the review, the search was limited to publications from the period 2015–2025. Data were analysed using Braun and Clarke (2006) thematic analysis. A hybrid deductive inductive approach was adopted; the three domains of the biopsychosocial model (sociocultural, psychological and biological) served as predetermined themes, while the sub-themes within each domain were generated inductively from the included studies. The thematic analysis was performed using Braun and Clarke’s six-phase guide as it offered the reviewer a systematic, reflective, thorough and robust framework that can be used when researching across various research traditions (Braun and Clarke, 2014). Understanding that the intended audience of this review also consists of individuals who are not part of academic communities, the Braun and Clarke’s thematic analysis guide allowed sophisticated analysis of data while ensuring such information is accessible and comprehensible to those who are not part of academic communities (Braun and Clarke, 2014; Maguire and Delahunt, 2017). The six steps are as follows (Naeem *et al.*, 2023):

1. *Become familiar with the data:* Familiarized myself with the research articles by reading and carefully re-reading the articles and noting initial ideas.
2. *Generate initial codes:* Created initial codes to describe the content found in research articles, and collated data that was relevant for each code.
3. *Search for sub-themes*: Collated related codes into candidate sub-themes and allocated each sub-theme to one of the three predetermined biopsychosocial themes.
4. *Review sub-themes*: Evaluated whether the sub-themes effectively reflected the codes and fitted the predetermined theme to which they had been allocated.
5. *Define sub-themes*: Named and described what each sub-theme entailed.
6. *Write-up*: Sub-themes were charted under their predetermined themes to visually illustrate their prominence. By immersing oneself in the data, these sub-themes were based on the familiarization and repetition amongst the studies included in this review.

## Inclusion/exclusion criteria

Upon identifying relevant studies in the field of GBV, the studies were scrutinised based on specific inclusion and exclusion criteria. Inclusion criteria focused on any existing literature from all study designs (quantitative, qualitative and mixed-methods studies) and publications in peer-reviewed journals related to biopsychosocial aetiologies of GBV among convicted South African men. Conversely, exclusion criteria filtered out studies not focused on biopsychosocial aetiologies of GBV among convicted South African men. To determine whether a study should be included or excluded in this narrative review, the reviewer used the following criteria (see Table 1).

**Table 1:** Eligibility criteria/inclusion criteria

Inclusion criteria	Exclusion criteria
Articles available in full text	Studies not focussing on men
Articles in English	Studies published before 2015
Studies focussing on men (population) Studies focussing on the biopsychosocial aetiologies of GBV (concept)	Studies not available in full text Studies published in languages other than English
Studies conducted in South Africa (context) Studies published between 2015 and 2025	Studies conducted outside South African context
Sources of evidence from any existing literature from all study designs	Studies that do not focus on the socio-cultural aetiologies of GBV

### Study selection

There were 1,235 potentially relevant citations found in the search conducted which were initially imported into the file folder for storage and citation management. On critical appraisal, 1,103 articles were excluded at title and abstract because the data was not reported in a relevant form or relevant data was not obtained from those articles, and others had an unclear case definition and data. Therefore, following duplication and relevance screening, the reviewers identified and removed 71 duplicate entries. Sixty-one citations were found to meet the selection criteria based on title and abstract, and the corresponding full-text articles. These were then transferred to the folder titled “Included folder” to facilitate the scoping review process. The reviewer conducted the full-text screening based on predefined inclusion and exclusion criteria. A total of 28 studies were excluded for the following reasons: 9 did not focus on men; 18 addressed GBV but did not focus on biopsychosocial aetiologies of GBV; eight were not published before 2015 period; and seven full texts were not conducted within the South African context. Ultimately, 33 studies met the inclusion criteria and were retained for data extraction and analysis.

## Quality appraisal

To assess the methodological quality and rigour of included studies, the reviewer used the Mixed-Method Appraisal Tool (MMAT), version 2018, as the primary quality assessment tool (Hong *et al.,* 2018). The reviewer used the MMAT for evaluating the methodological quality of reviewed studies (qualitative, quantitative and mixed-method studies) (Hong *et al.*, 2018). This study applied the MMAT appraisal tool to evaluate whether studies intended for inclusion had a clearly defined research question and whether collected data would help address the research question. The reviewer also assessed qualitative articles to determine whether the qualitative approach used in the study was appropriate for answering the research question, and to make sure the conclusions drawn from the qualitative data were accurate. Additionally, the reviewer assessed whether the interpretation of the research findings was sufficiently supported by the data collected, and whether there was a logical link between the qualitative sources of data collection, analysis, and interpretation. The reviewer evaluated mixed-methods articles for their appropriateness in answering the research question and to determine whether qualitative and quantitative components were integrated to address the question. In addition, the reviewer assessed whether qualitative and quantitative components were described correctly, whether discrepancies were addressed and whether the qualitative and quantitative aspects of the study supported the quality criteria for each practice of the involved method (Hong *et al.*, 2018).

## Ethical considerations

This study used a desktop review methodology, involving the collection and analysis of data from already published sources. Since no human participants were involved, this review did not require ethics approval under standard guidelines for research involving human subjects. However, this desktop review was conducted as a component of a larger primary research project that involved empirical data collection. The broader project received ethical approval from the College of Human Sciences Research Ethics Committee (CREC) at the University of South Africa, with reference number: Rec-240816-052. This ethical approval covers the overall research activity, including the desktop review as part of the primary study. Therefore, the desktop review itself did not require separate ethical approval but was conducted within the approved framework of the larger project.

## Findings

In line with the biopsychosocial framework, the findings are organised under three predetermined themes:

1. sociocultural perspective;
2. psychological perspective; and
3. biological perspective.

The sub-themes within each theme were generated inductively from the reviewed literature (Table 2). The sociocultural theme is presented first because it provides the broader context for GBV in South Africa. Biological and psychological risk factors, such as elevated testosterone or childhood maltreatment, are found among men in every country; what distinguishes South Africa, with its extraordinarily high rates of GBV, is its brutal legacy of colonialism and apartheid and the structural inequality, gender norms and normalised violence that this legacy has left behind. The psychological and biological sub-themes are therefore best read as mechanisms that operate within, and are amplified by, this sociocultural context.

**Table 2:** Predetermined themes and constructed sub-themes

Predetermined themes	Constructed sub-themes
1. Sociocultural perspective	History of colonialism and the apartheid system Patriarchal culture Adherence to negative ideas of masculinity Inequitable gender norms Poverty
2. Psychological perspective	Attachment Mentalising capacities Emotional regulation Childhood experiences Alcohol/drug use experiences
3. Biological perspective	Genetics Neurotransmitters Lower level of serotonin Dopamine Brain injury

### Theme 1: Sociocultural perspective

From the sociocultural perspective, evidence suggested that the history of colonialism and the apartheid system, patriarchal culture, adherence to negative ideas of masculinity, inequitable gender norms and poverty are factors that legitimise GBV (Graaff and Heinecken, 2017; Government of South Africa, 2020; Uysal and Gizem, 2020).

*Sub-theme 1: History of colonialism and the apartheid system:* The literature consistently locates the roots of contemporary GBV in the structural violence of colonial and apartheid rule, which shaped how South African men came to construct and enact their gender identities (Pyke, 2020; Van Niekerk and Boonzaier, 2015). The colonial legacy embedded patriarchal values in South African society and caused women to be subordinate (Hoosain and Robertson, 2023). Colonialism was detrimental to gender equalities that existed in African countries such as South Africa prior to colonial inception and brought the concept that women should stay at home and leave the real work to be performed by men (Mikidady, 2022). Colonisation and government assimilation policies have disrupted traditional indigenous families (Wei *et al.*, 2024). Through a desire to control African women, the colonial state created a range of systems, including the restriction of movement and the regulation of marriage, which reinforced the authority of African elders and men. Customary wives’ rights to live somewhere, or to access a job or a house, came to depend on their marital relationship, severely compromising women’s choices about marriage (Moore, 2020). Within the process of colonisation, men rapidly acquired literacy and quickly found their way into white-collar employment (Ntshangase and Tlhakodisho, 2022). When accessing education, African boys were preferred to girls; thus, boys, who were the future men, became more educated than girls. It is from these relations that colonisers implanted into African lifeways that the current gender inequalities have evolved (Mikidady, 2022). Formal sectors during the colonial era and after independence were primarily reserved for men, while women were engaged in informal work (Montgomery, 2017). Other studies have highlighted the socioeconomic and political transformation induced by the historical shock of colonialism as the primary driver of historic gender inequality in Africa. Gender power inequities, the lower social status of women and surrounding social norms that perpetuate social, economic and political inequality are considered to be the root causes of violence (Treves-Kagan *et al.*, 2020). For example, cultures that support traditional gender norms and gender inequality, and the idea that women should stay home and be submissive, while men should make the decisions and provide for the family, encourage men to be violent towards their partner if the partner is not submissive (Winner, 2023). Those cultures encourage the idea that “women are not equal to men” (Winner, 2023).

*Sub-theme 2 – Patriarchal culture*: Sociocultural theory posits that domestic violence, rooted in patriarchy, is male aggression towards women, and is solely the product of the socially sanctioned dominance and control over women by men, and that violence is used tactically as a form of power and control (Corvo, 2019). Patriarchy is so deeply entrenched in African societies that many men and women consider it to be a normal way of life (Ademiluka, 2018). Research suggests that dominant ideologies teach children from a young age that women and girls should assume caring roles such as raising children and performing household tasks, while men’s identity should be defined by their individuality, their ability to take risks and their ability to earn an income and provide for the family (Kedde *et al.*, 2018). Men are stereotyped as being the primary breadwinners of the household, sexually driven, expected to be physically strong or tough and using violence to control others (Graaff and Heinecken, 2017). Combining these factors often leads to men resorting to violence and using aggression to solve problems and resolve confrontations (Graaff and Heinecken, 2017). The evidence suggests that patriarchal norms serve as a foundation for violent behaviours in intimate and social contexts.

*Sub-theme 3 – Adherence to negative ideas of masculinity:* GBV is more prevalent in societies with rigid gender roles or in patriarchal communities in which male dominance is engrained in a masculine identity (Graaff and Heinecken, 2017). The gendered risk factors for GBV perpetration include a strong patriarchal belief about gender roles, power and control; objectification of women; and a feeling of entitlement to respect and sex (Rousseau-Jemwa and Hendricks, 2016). Additional norms that contribute to the acceptance of GBV include thinking that a man is the authority in a household, that a husband “owns” his wife, or that a woman is not able to refuse to have sex with a man who provides for her financially, and these can then provide justifications for the use of violence (Graaff and Heinecken, 2017). The practice of bride price, for instance, may reinforce a husband’s sense of ownership, leading him to feel justified in mistreating his wife or becoming angry if she does not fulfil expected roles such as cleaning, cooking, childcare or providing sex (Graaff and Heinecken, 2017). Thus, GBV can be understood as one context in which men attempt to express socialised masculine beliefs and norms (Berke *et al.*, 2019). This demonstrates that cultural ideals of masculinity are central to understanding GBV perpetration.

*Sub-theme 4 − Inequitable gender norms:* Gender power inequities, or the culturally constructed understandings of how men and women are supposed to behave implicitly, and explicitly in many cultures, condone violence against women (Treves-Kagan *et al.*, 2020). Furthermore, boys who grow up with the mentality that they are superior solely because of their gender tend to become violent towards their female partner when they feel that their partner is not obedient enough, and their superiority as males is being compromised (Winner, 2023). Consequently, the social construct of masculinity in patriarchal societies leads to the oppression and control of women by men, leading to male perpetration and women’s vulnerability (Mngoma *et al.*, 2016). These pervasive gender norms promote the use of violence as acceptable social practices and feed into the ideas of masculinity that are centred on male control of women, male sexual entitlement, inequitable gender attitudes, risk taking and antisocial behaviour, as well as ideas about femininity that expect women to acquiesce to male partners’ sexual desires (Government of South Africa, 2020). This underscores how societal expectations about gender reinforce violence and maintain women’s subordinate status, contributing to ongoing gender inequalities.

*Sub-theme 5 − Poverty:* The levels of unemployment have increased, the income gap has grown and the resultant inability of many men to achieve the hegemonic norms of being an economic provider may contribute to their use of violence as a reaction (Graaff and Heinecken, 2017). Today, cultural and traditional expectations of manhood remain aligned with the key masculine traits of strength, virility, the ability to withstand pain and to provide for and protect their family (Pyke, 2020). Because the provider role is often linked to being employed or having land or livestock, unemployment or an inability to meet social expectations of “successful” manhood may trigger a crisis of male identity (Graaff and Heinecken, 2017). Where men are unable to maintain masculine superiority, they are more likely to use violence to exert power (Government of South Africa, 2020). This highlights the role of economic stress and poverty as significant contributors to GBV, linking financial insecurity with increased risk of violence rooted in traditional gender expectations.

### Theme 2: Psychological perspective

From the psychological perspective, evidence suggested that individual psychopathology or dysfunctional personality structures, which are learnt and shaped by early childhood experiences, contribute to GBV predisposition in men (Grover, 2015). The urge for aggression is also learnt in the socialisation process of the individual (attachment, mentalising capacities, emotional regulation and childhood experiences) (Uysal and Gizem, 2020; Corvo *et al.*, 2018; Taubner *et al.*, 2017; Berke *et al.*, 2019; Winner, 2023). This indicates that early developmental experiences critically impact later behavioural patterns, contributing to violent tendencies. However, the construction of masculinities, femininities and violence does not occur in a vacuum and should be understood within its broader context (Van Niekerk and Boonzaier, 2019); the psychological pathways described below therefore unfold within the sociocultural conditions outlined in Theme 1.

*Sub-theme 1 – Attachment*: Early childhood adversities and disrupted relationships contribute to the development of pathological aggression and violence (Yakeley, 2018). Attachment styles developed during child–parent relationships subsequently extend to later relationships in adulthood, given the parallels between these relationships (Shen *et al.*, 2021). Thus, much of the basis of how individuals interact with the world around them has to do with how they experience relationships with others very early in life (Golding and Fitzgerald, 2019). Early-life disruptions in healthy attachment caused by erratic, violent or unresponsive caregivers, which influence individuals throughout their life span, may interact with subsequent psychosocial risk or stressors to produce personality disorders and more severe violence (Corvo, 2019). Violence and abuse could be the result of a dysfunctional attachment to others (Rogers and Ali, 2023). This underscores the importance of healthy early relationships in preventing the development of violent behaviours later in life.

*Sub-theme 2 – Mentalising capacities* (the ability to understand one’s own and others’ mental states (Taubner *et al.*, 2017): Mentalising is the capacity to understand ourselves and others in terms of intentional mental states, such as feelings, desires, wishes, attitudes and goals (Luyten and Fonagy, 2015). Chronic violence can be regarded as an expression of the individual’s inability to symbolise or mentalise feelings and thoughts (Taubner *et al.*, 2017). Mentalising can be severely impaired when early relationships become brutalised (Taubner *et al.*, 2017). If a person is able to mentalise from an early age, that person has begun to develop some consciousness about their own emotions and the emotions of those with whom they interact (Golding and Fitzgerald, 2019). Violent individuals frequently experience a merely teleological mode of understanding (which is a way in which we ordinarily understand others’ intentional actions) that is strictly limited to detecting what happens in the physical world, that is, what is the outcome of an action, regardless of its impact on subjective experience (of either the self or the other) (Taubner *et al.*, 2017; Perner *et al.*, 2018). Being recognised as a feeling and thinking human being is a prerequisite to experiencing oneself and others as thinking and feeling beings, and to be able to fully mentalise others (Taubner *et al.*, 2017). Growing up in an environment of maltreatment (discontinuous relationships) can prevent an individual from being able to check out, to correct or confirm his gut reaction to the ambiguous hostile intent he may encounter later in adolescence and adulthood (Golding and Fitzgerald, 2019). This highlights how early relational trauma can hinder emotional understanding and contribute to violent behaviour.

*Sub-theme 3 – Emotional regulation:* GBV perpetration may function as a means of regulating distressing emotions stemming from pressures to conform to precarious masculine norms. Therefore, aggression may confer a mood-regulating function (Berke *et al.*, 2019). Early experiences of trauma, neglect and loss have an impact on the attachment process by interfering with the normal development of emotional or affect recognition and expression, empathy and impulse control (Yakeley, 2018). Maltreatment experienced early has been described as a way in which children are toughened and come to maintain their physical aggressiveness (Golding and Fitzgerald, 2019). Prevailing gender norms governing emotional expression hold that men should suppress their emotions, with the exception of anger (Berke *et al.*, 2019). As such, emotional avoidance and suppression may serve as a strategy for nonconforming men to reassert alignment with their precarious masculine status and to manage fear and the threat of social condemnation (Berke *et al.*, 2019). This revealed the role of social and emotional conditioning in perpetuating violence.

*Sub-theme 4 – Childhood experiences*: Children exposed to violent environments, such as witnessing domestic violence between parents, experiencing poor parenting and experiencing physical discipline, are at greater risk of becoming violent adults (Winner, 2023). Violence can be normalised for individuals who witness this situation in social structures where violence is considered ordinary (Uysal and Gizem, 2020). Research found that witnessing domestic violence as a child or experiencing childhood physical or sexual violence is associated with re-victimisation as well as perpetration as an adult (Treves-Kagan *et al.*, 2020). These findings emphasise the importance of early intervention and the impact of social context on violent behaviour.

*Sub-theme 5 – Alcohol/drug use experiences:* Alcohol and drug use increase the likelihood of violent behaviour, particularly in intimate relationships (Maphakela *et al.*, 2026). The majority of GBV cases occur after consumption of drugs and alcohol at the taverns and shebeen (places where alcohol sales are made), leading to loss of control, aggression and fights (Maphakela *et al.*, 2026). From a global health standpoint, substance use is widely recognised as a key modifiable risk factor for violence, as alcohol-related disinhibition and drug-induced impulsivity reduce behavioural control and increase aggressive responses to conflict or perceived disrespect (Ani, 2026). In the South African context, alcohol consumption is socially normalised, particularly in informal settlements and among marginalised men, and these risks are further intensified by poverty, unemployment and chronic social stress (Ani, 2026). Individuals who struggle with both drug or alcohol use and aggressive behaviour are more likely to behave irresponsibly, put themselves or others at risk and become entangled in the legal system (Tomlinson *et al.*, 2016). The evidence indicates that substance use significantly increases the likelihood of violent behaviour, emphasising the need for targeted interventions addressing substance-related risks to reduce GBV.

### Theme 3: Biological perspective

Existing literature shows that violent behaviour such as GBV is perpetuated by multiple and complex mechanisms in the individual, community and societal spheres (Mngoma *et al.*, 2016). From a biological perspective, the risk of adult domestic violence perpetration is underpinned by genetic vulnerabilities where GBV has been linked to evolutionary roots (genetic), centres on neurotransmitters, lower levels of serotonin, dopamine and brain injury (Chester and DeWall, 2018; Corvo and Dutton, 2015; Rogers and Ali, 2023; World Health Organization, 2017).

However, much of the evidence in the below sub-themes were derived from international studies, and there is a notable gap regarding the specific biological correlates of violence among convicted men in South Africa. Consequently, these findings should be viewed as indicative of possible mechanisms that warrant further local investigation to confirm these mechanisms among South African men. Moreover, biological risk factors such as elevated testosterone, low serotonin or particular gene variants are present among men in every society and cannot, on their own, account for South Africa’s exceptionally high rates of GBV. Their relevance lies in how they interact with the adverse environmental conditions described in Themes 1 and 2, as the gene–environment interactions discussed below illustrate.

*Sub-theme 1 – Genetics*: GBV, such as intimate partner violence (IPV), has evolutionary roots and is transmitted from parents to their children, with clear genetic underpinnings (Chester and DeWall, 2018). For instance, having a specific gene, like the low-activity allele of the monoamine oxidase-A gene (MAOA-L), is associated with antisocial behaviour in both males and females when combined with adverse environmental conditions such as receiving abusive or neglectful caregiving early in life (Golding and Fitzgerald, 2019). Epigenetic mechanisms (biological processes that regulate gene expression without altering the DNA sequence) have even been found to be the specific genes that predispose individuals to antisocial acts (e.g. IPV), which exhibit more methylation (i.e. an epigenetic mechanism) if the mother experienced IPV in utero (Chester and DeWall, 2018). Such epigenetic mechanisms are concrete pathways through which biological and environmental forces interact to influence aggressive or violent behaviour (Chester and DeWall, 2018). While these studies collectively suggest a biological basis, the extent to which these mechanisms operate among men in South Africa remains underexplored, indicating a gap that future research should address.

*Sub-theme 2 – Neurotransmitters:* When levels of the hormones and neuromodulators cortisol and testosterone are, depleted or elevated, respectively, a synergistic effect on social aggression and violence occurs (Corvo and Dutton, 2015). Testosterone affects aggression by influencing the development of various areas of the brain that control aggressive behaviours (Chester and DeWall, 2018). High testosterone levels in men are associated with greater verbal and physical aggression, dominance, sensation seeking and antisocial behaviours, including criminal activity (Rogers and Ali, 2023). However, most evidence stems from Western populations, highlighting a gap regarding biological differences in South African contexts that future research should explore.

*Sub-theme 3 – Serotonin:* A lower level of serotonin is also identified as predictive of impulsive and violent behaviour (Rogers and Ali, 2023). Low serotonin levels have been found to have a negative effect on mood and behaviour, whereas increased serotonin levels have been found to result in improved social interaction and decreased aggression (Rogers and Ali, 2023). Specifically, serotonin inhibits aggression, and future aggression has been predicted by low serotonin levels (Jhangiani, 2022). Violent criminals have lower levels of serotonin than nonviolent criminals, and criminals convicted of impulsive violent crimes have lower serotonin levels than criminals convicted of premeditated crimes (Jhangiani, 2022). Serotonin plays a major role in regulating mood and emotion, and in some aspects of executive functioning (Corvo and Dutton, 2015). Furthermore, serotonin is associated with social behaviour, increased sensitivity to social factors and empathy (Corvo and Dutton, 2015).

*Sub-theme 4 – Dopamine:* Dopamine also plays a major role in the initiation and enactment of aggression (Corvo and Dutton, 2015). Dopamine levels significantly influence motivation, pleasure-seeking, perseverance, addiction and working memory (Corvo and Dutton, 2015). Research has identified three key dopaminergic genes (DAT1, DRD2 and DRD4) that are linked to violence perpetration and the processing of dopamine (Schwab-Reese *et al.*, 2020). Studies indicate that the DAT1 gene affects dopamine reuptake, with the 10-repeat allele linked to increased violence and lower self-control in young men due to impaired impulse control (Schwab-Reese *et al.*, 2020). Additionally, the DRD2 and DRD4 genes are associated with violence perpetration, where the DRD2 A1 allele is associated with increased susceptibility to environmental triggers, elevated response to stressful situations and an increased propensity for violence perpetration, while the DRD4 7-repeat allele correlates are associated with reduced response to dopamine, poor impulse control and increased odds of violence perpetration (Schwab-Reese *et al.*, 2020). Notably, Schwab-Reese *et al.* (2020) examined these genes in interaction with financial stressors, underscoring that genetic risk is expressed in relation to social and economic conditions such as those described under the poverty sub-theme.

*Sub-theme 5 – Brain injury:* Brain injury has been linked to aggression and is a unique predictor of IPV perpetration (Bannon *et al.*, 2015). Of all the brain injuries, traumatic brain injury (TBI) survivors mainly suffer from disturbances related to aggression and impulsiveness, which not only affect them but also their family and friends (Ahmed *et al.*, 2017). The correlations of head injury that may predispose brain-injured individuals to violence are executive functioning deficits, which may serve as the mediator through which violence occurs after a TBI (Bannon *et al.*, 2015). Common sequelae of a head injury or reduced executive functioning may include difficulty regulating emotions and problem-solving, not understanding social cues, lacking insight into behaviour and being self-centred, aggressive, impulsive and easily irritated (Lansdell *et al.*, 2022). Survivors of brain injuries face difficulties with behaviour, anger management and self-monitoring, whereas partners and family members of survivors of brain injuries observe changes in the survivor’s personality, irritability, rage outbursts and reduced impulse control as long-term consequences of head injury (Rogers and Ali, 2023). The severity and behavioural consequences of TBI appear consistent across studies, yet there is limited research examining the prevalence and impact of TBI among men in South Africa. This gap suggests that while biological mechanisms such as brain injury may be universally relevant, contextual factors unique to South Africa could modulate these relationships.

## Discussion

This review set out to identify the reported biopsychosocial aetiologies of GBV among convicted South African men. Organising the literature under the three predetermined domains of the biopsychosocial model showed that, unlike earlier formulations that treated the intergenerational transmission of domestic violence purely as learnt behaviour (Corvo, 2019), GBV is better understood as the product of interacting sociocultural, psychological and biological influences (Fleming *et al.*, 2015; Grover, 2015; Lehman *et al.*, 2017). The central argument that emerges, however, is that these domains do not carry equal weight in explaining South Africa’s situation. Risk factors such as elevated testosterone, low serotonin, brain injury or insecure attachment are found among men everywhere, yet South Africa faces extreme levels of GBV (Graaff and Heinecken, 2017). It is the country’s sociocultural history that best accounts for this difference, and it is within that history that the psychological and biological findings should be interpreted.

The sociocultural findings suggest that colonialism and apartheid did not simply coexist with GBV but actively produced the conditions for it. Colonial rule entrenched patriarchal values, disrupted families and tied women’s livelihoods and residence to marriage and male authority (Hoosain and Robertson, 2023; Moore, 2020; Wei *et al.*, 2024), while unequal access to education and formal employment institutionalised men as dominant wage earners and women as household caretakers (Allen, 2018; Mikidady, 2022). These arrangements have outlived the regimes that created them. They persist in gender norms that equate manhood with control over women, sexual entitlement and the provider role (Government of South Africa, 2020; Graaff and Heinecken, 2017), and they are so embedded that patriarchy is widely experienced as normal (Ademiluka, 2018). An important implication is that GBV in South Africa is not only an expression of male dominance but, in some cases, a response to its perceived loss: where high unemployment and inequality prevent men from achieving the provider ideal, violence may become an alternative means of asserting masculinity (Graaff and Heinecken, 2017). This may help to explain why progressive legislation has not, on its own, reduced GBV, since laws address acts of violence rather than the structural and normative conditions that sustain them.

The psychological findings describe the pathways through which this social history is carried into individual lives. Exposure to domestic violence, harsh physical discipline and maltreatment in childhood predicts later perpetration (Treves-Kagan *et al.*, 2020; Winner, 2023), at least partly by disrupting attachment, mentalising and emotional regulation (Corvo, 2019; Taubner *et al.*, 2017; Yakeley, 2018). Deficits in emotion regulation arising from such adversity may in turn manifest as behavioural problems linked to GBV perpetration in adult relationships (Bliton *et al.*, 2016; Hyde-Nolan and Juliao, 2012). In a society where violence has long been part of social relations, such exposure is not an exceptional misfortune but a common developmental experience through which violence becomes normalised (Uysal and Gizem, 2020; Van Niekerk and Boonzaier, 2015). The findings on emotional regulation are particularly telling that norms that permit men to express anger but not vulnerability (Berke *et al.*, 2019) mean that the psychological consequences of early adversity are channelled through a culturally sanctioned script of masculine aggression. Substance use acts as a proximal trigger within this configuration, and its effects are intensified by poverty, unemployment, chronic social stress and the normalisation of heavy drinking in marginalised communities (Ani, 2026; Maphakela *et al.*, 2026). Psychological risk factors are thus interwoven with, rather than independent of, the sociocultural context.

The biological evidence should be interpreted in the same light. Genetic variants, hormonal and neurotransmitter profiles and brain injury are associated with aggression and IPV perpetration (Bannon *et al.*, 2015; Chester and DeWall, 2018; Rogers and Ali, 2023), but the most informative findings concern gene–environment interactions. The MAOA-L allele is linked to antisocial behaviour chiefly when combined with abusive or neglectful early caregiving (Golding and Fitzgerald, 2019), dopaminergic genes have been studied in interaction with financial stressors (Schwab-Reese *et al.*, 2020), and epigenetic changes have been associated with in utero exposure to IPV (Chester and DeWall, 2018). Biological vulnerabilities are therefore likely to be expressed more often in environments marked by childhood adversity, economic stress and normalised violence. Given the limited local research on TBI, and its association with aggression and impaired impulse control (Ahmed *et al.*, 2017), screening for brain injury among convicted men may also be warranted. Because this evidence is drawn largely from international studies, however, these mechanisms remain indicative rather than established for South African men.

Taken together, the findings suggest a layered model: sociocultural conditions rooted in colonialism and apartheid set the stage, psychological processes formed through early adversity translate those conditions into individual dispositions and biological factors may modulate how strongly those dispositions are expressed. For practitioners working with men convicted of GBV offences, this implies that assessment and intervention focused solely on the individual are unlikely to be sufficient without attention to the gender norms and structural conditions that shaped his behaviour (Peacock and Barker, 2014; Walton *et al.*, 2017).

## Limitations

This study had the following potential limitations: the findings primarily pertain to men in South Africa and may not be generalisable to other settings. While statistical data indicate that men are frequently identified as perpetrators of GBV, it is crucial to interpret these figures within a broader systemic context. Crime data are subject to limitations, including underreporting. While the literature provides valuable insights into the biological, psychological and sociocultural factors associated with GBV among convicted men in South Africa, there remains a notable gap in critically synthesising the biological factors’ findings. Literature specific to the South African context is limited, and what exists often generalises findings from international studies. As noted under Theme 3, the biological findings should therefore be treated as provisional. Future research should address this gap by exploring how biological factors like testosterone interact with local sociocultural norms and structural vulnerabilities unique to South Africa. The review primarily relies on published literature, which may introduce a publication bias wherein studies with significant findings are more likely to be published than those with null or inconclusive results.

## Conclusion

In conclusion, this review underscores that, despite progressive legislation and intervention programmes, the prevalence of GBV in South Africa remains alarmingly high. Using the biopsychosocial framework as an organising lens, the review shows that sociocultural, psychological and biological factors are interconnected in shaping violent behaviour among convicted South African men, but that they do not carry equal explanatory weight. South Africa’s legacy of colonialism and apartheid, and the patriarchal norms, inequality and normalised violence it has left behind, provide the context that distinguishes the country’s exceptionally high rates of GBV, while psychological and biological factors act as pathways and modifiers within that context. This offers insights for policy, practice and future research, and supports an integrated approach to understanding and addressing GBV in forensic mental health settings.

## Recommendation

This review’s originality lies in its effort to inform psychologists, healthcare providers and professionals from various disciplines, including probation officers, police officers, social services officials and policymakers who work closely with men convicted of GBV offences. It emphasises the application of the biopsychosocial model to enhance risk assessments, therapeutic interventions and treatment programmes within South African correctional services, with the ultimate goal of reducing GBV. Recognising these complex, multilayered influences underscore the need for holistic intervention strategies that go beyond punitive measures to tailored rehabilitation programmes that incorporate biological, psychological and sociocultural screening, trauma-informed care and gender-transformative interventions within correctional facilities. Such efforts could reduce recidivism, foster gender equality and support the rehabilitation of offenders, ultimately contributing to more effective and sustainable policies for managing GBV in forensic contexts and advancing broader rehabilitation programmes initiatives within correctional facilities. It was therefore imperative that this phenomenon be studied in more depth and understood in context and time, because this remains an area that requires more research.

## Data Availability

The data that support the findings of this study are available from the corresponding author upon reasonable request. All relevant materials and resources used in this research have been appropriately cited and can be accessed through the links in the reference list.

## Declarations

The author(s) confirm(s) that this article adheres to the ethical standards set forth by *International Journal of Prison Health*. The research involved no human or animal participants and complied with all relevant ethical guidelines.
